# Global birth prevalence of congenital heart defects 1970–2017: updated systematic review and meta-analysis of 260 studies

**DOI:** 10.1093/ije/dyz009

**Published:** 2019-02-19

**Authors:** Yingjuan Liu, Sen Chen, Liesl Zühlke, Graeme C Black, Mun-kit Choy, Ningxiu Li, Bernard D Keavney

**Affiliations:** 1 Division of Cardiovascular Sciences, School of Medical Sciences, Faculty of Biology, Medicine and Health, The University of Manchester, Manchester, UK; 2 Department of Social Medicine, West China School of Public Health, Sichuan University, Chengdu, China; 3 Department of Paediatrics, Red Cross War Memorial Children’s Hospital, University of Cape Town, Cape Town, South Africa; 4 Division of Cardiology, Department of Medicine, Groote Schuur Hospital and University of Cape Town, Cape Town, South Africa; 5 Manchester University NHS Foundation Trust, Manchester Academic Health Science Centre, Manchester, UK; 6 Division of Evolution & Genomic Sciences, School of Biological Sciences, Faculty of Biology, Medicine and Health, The University of Manchester, Manchester, UK

**Keywords:** Congenital heart disease, prevalence, meta-analysis, systematic review, geographical region, national income

## Abstract

**Background:**

Globally, access to healthcare and diagnostic technologies are known to substantially impact the reported birth prevalence of congenital heart disease (CHD). Previous studies have shown marked heterogeneity between different regions, with a suggestion that CHD prevalence is rising globally, but the degree to which this reflects differences due to environmental or genetic risk factors, as opposed to improved detection, is uncertain. We performed an updated systematic review to address these issues.

**Methods:**

Studies reporting the birth prevalence of CHD between the years 1970–2017 were identified from searches of PubMed, EMBASE, Web of Science and Google Scholar. Data on the prevalence of total CHD and 27 anatomical subtypes of CHD were collected. Data were combined using random-effect models. Subgroup and meta-regression analyses were conducted, focused on geographical regions and levels of national income.

**Results:**

Two hundred and sixty studies met the inclusion criteria, encompassing 130 758 851 live births. The birth prevalence of CHD from 1970–2017 progressively increased to a maximum in the period 2010–17 of 9.410/1000 [95% CI (confidence interval) 8.602–10.253]. This represented a significant increase over the fifteen prior years (*P* = 0.031). The change in prevalence of mild CHD lesions (ventricular septal defect, atrial septal defect and patent ductus arteriosus) together explained 93.4% of the increased overall prevalence, consistent with a major role of improved postnatal detection of less severe lesions. In contrast the prevalence of lesions grouped together as left ventricular outflow tract obstruction (which includes hypoplastic left heart syndrome) decreased from 0.689/1000 (95% CI 0.607–0.776) in 1995–99, to 0.475/1000 (95% CI 0.392–0.565; *P* = 0.004) in 2010–17, which would be consistent with improved prenatal detection and consequent termination of pregnancy when these very severe lesions are discovered. There was marked heterogeneity among geographical regions, with Africa reporting the lowest prevalence [2.315/1000 (95% CI 0.429–5.696)] and Asia the highest [9.342/1000 (95% CI 8.072–10.704)].

**Conclusions:**

The reported prevalence of CHD globally continues to increase, with evidence of severe unmet diagnostic need in Africa. The recent prevalence of CHD in Asia for the first time appears higher than in Europe and America, where disease ascertainment is likely to be near-complete, suggesting higher genetic or environmental susceptibility to CHD among Asian people.

**Key Messages**

- This meta-analysis incorporated global data on prevalence of congenital heart disease (CHD) from 260 studies (130 758 851 births), of which 142 (103 732 049 births) were published after 2010.
- The prevalence of ‘mild lesions’ [ventricular septal defect (VSD), atrial septal defect (ASD) and patent ductus arteriosus (PDA)] in particular, increased throughout the study period, probably reflecting improved detection. The prevalence of left ventricular outflow tract obstruction (LVOTO) was decreased since 1995, possibly reflecting increased prenatal diagnosis and consequent termination of pregnancy.
- Regional differences in CHD prevalence were identified. The average CHD prevalence reported from Africa was the lowest (overall and mild lesions), whereas that from Asia was the highest (mild lesions).
- The prevalences of ASD (reversely) and LVOTO (positively) were both correlated with gross national incomes (GNIs).
- Overall, the reported birth prevalence of CHD globally is increasing, raising important questions regarding healthcare resource allocation to care for this complex patient group.

## Introduction

Congenital heart disease (CHD) is the commonest birth defect worldwide, affecting millions of newborns every year.^1^ CHD is typically defined as a structural abnormality of the heart and/or great vessels that is present at birth. Although approximately 20% of CHD incidence can be attributed to genetic syndromes, teratogen exposure or maternal diabetes, there remains substantial uncertainty regarding risk factors for the remaining 80% of cases.^2^ Improved medical and surgical care have transformed the prognosis for children born with severe CHD in developed countries, but in parts of the developing world, access to treatment for the severer conditions remains unavailable. Indeed, the birth prevalence of CHD across the world is not yet accurately established, potentially obscuring differences in environmental and/or genetic risk factors for CHD between regions that could have public health consequences.

A systematic review of global prevalence data since 1930 was conducted by van der Linde *et al.*^1^ in 2010, suggesting an increase in CHD prevalence from the 1930s until 1995, with stabilization between the years 1995–2009. Marked heterogeneity between global regions was noted, chiefly attributed to differential availability of diagnostic medical technology. However, since that valuable report, the literature has significantly expanded, and the availability of diagnostic technology in some parts of the developing world has likely changed considerably in parallel with rapid economic development, prompting us to conduct an updated systematic review. In addition, the size of the available dataset in 2010 restricted van der Linde *et al.* to subgroup analysis of only the eight commonest CHD subtypes; we hypothesized that in a larger dataset, subgroup analysis of less common, and typically more severe, phenotypes would be possible to discern important prevalence trends.

## Methods

### Search strategy

We searched the literature with a cut-off date of June 2017, using PubMed, EMBASE, Web of Science and Google Scholar, for peer-reviewed studies with English abstracts which reported the birth prevalence of CHD anywhere in the world. The main search terms used were ‘congenital heart disease’, or ‘congenital heart defect’, or ‘heart abnormality’, or ‘heart malformation’, and ‘prevalence’, or ‘incidence’, or ‘frequency’, or ‘epidemiology’. We restricted ourselves to studies published after 1970, as this approximately coincides with the widespread adoption of echocardiography, the current standard diagnostic modality in paediatric cardiology.^3^

### Data review

Review for eligibility was carried out in three stages, of titles, abstracts and full texts. All review and data extraction was carried out independently by the same two authors (Y.L. and S.C.) and any discrepancy resolved by discussion. In short, original peer-reviewed studies reporting on overall CHD prevalence of populations between birth and age 6 years were included. Studies on specific populations which would cause bias in estimation, for example the children of women with diabetes, were excluded. The details of inclusion and exclusion criteria are shown in Supplementary Table 1, available as Supplementary data at *IJE *online. All included papers were evaluated with the Newcastle–Ottawa Scale (NOS, maximum 9 stars), which is a quality assessment tool recommended by Cochrane Collaboration and widely used for observational studies.^4–6^ Quality scores and list of included papers are presented in ‘Supplementary Data’, available as Supplementary data at *IJE *online.

Data on overall CHD prevalence, and prevalence of 27 CHD subtypes, were extracted (raw data and abbreviations of CHD subtypes are available in ‘Supplementary Data, available as Supplementary data at *IJE *online). In the principal analyses, CHD was classified into seven phenotypic categories, following the approach of Botto *et al.*^7^ which groups together lesions on the basis of their anatomical/developmental similarity (Supplementary Table 2, available as Supplementary data at *IJE *online). With respect to assigning lesion severity, we followed the modified classification of Hoffmann^8–10^ (Supplementary Table 3, available as Supplementary data at *IJE *online); ventricular septal defect (VSD), atrial septal defect (ASD) and patent ductus arteriosus (PDA) were grouped together as ‘mild’ lesions.

For time-trend analyses of CHD prevalence, studies were analysed in five-year groups dependent on year of investigation. In total, nine groups were defined: 1970–74, 1975–79, 1980–84, 1985–89, 1990–94, 1995–99, 2000–04, 2005–09 and 2010–17. The final group ‘2010–17’ contained more than 5 years since only three studies had data collected after 2014.

The gross national income per capita by country, calculated using the Atlas method, was collected from the database of the World Bank. Countries were classified as ‘Low’ (≤USD 995), ‘Lower-middle’ (USD 996–3895), ‘Upper-middle’ (USD 3896–12 055) and ‘High’ (≥USD 12 056) according to their gross national income.^11^

### Statistical analysis

Data were analysed using Rmeta and STATA 14. Random effect models were applied. The association between prevalence of CHD and geographical regions and income levels were calculated by meta-regression or subgroup analyses. In subgroup analyses, the false discovery rate-adjusted *P*-values using Benjamini–Hochberg procedures were applied for multiple comparisons. The significance level was defined as (two-tailed) *P* < 0.05. Data are presented as means with 95% confidence intervals.

## Results

**Figure 1.**
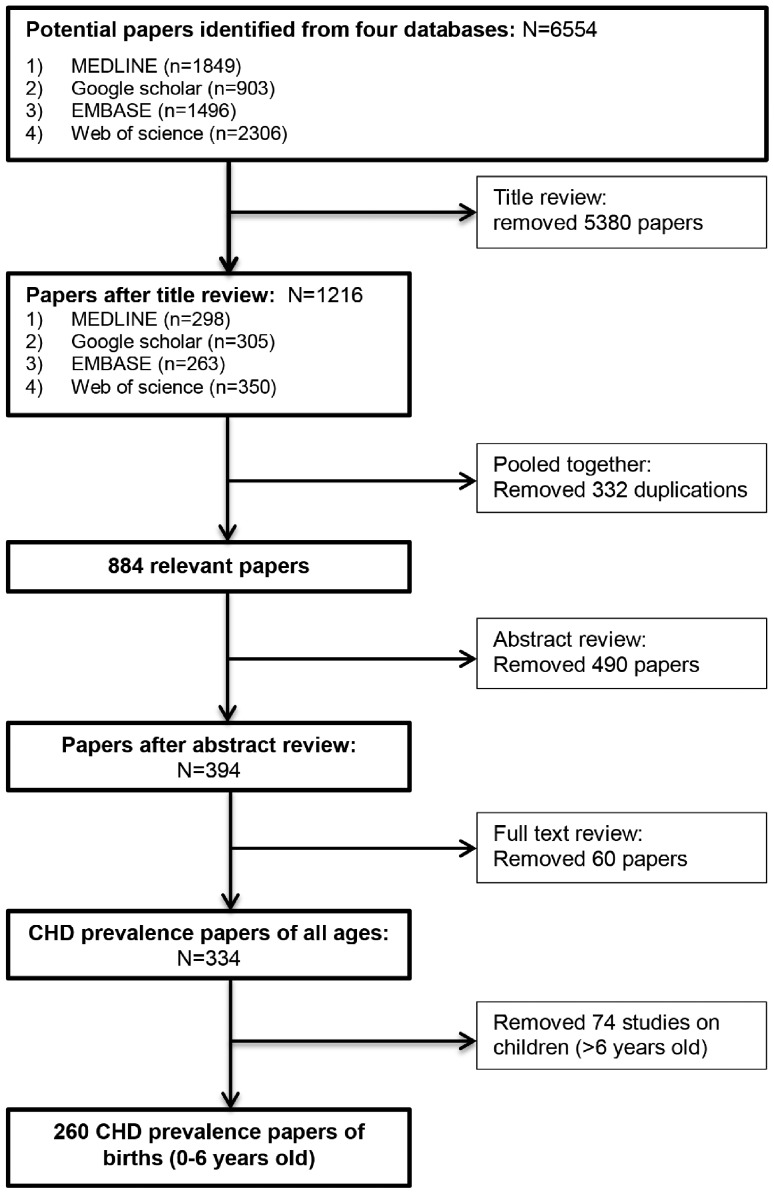
PRISMA flow diagram for review.

**Figure 2.**
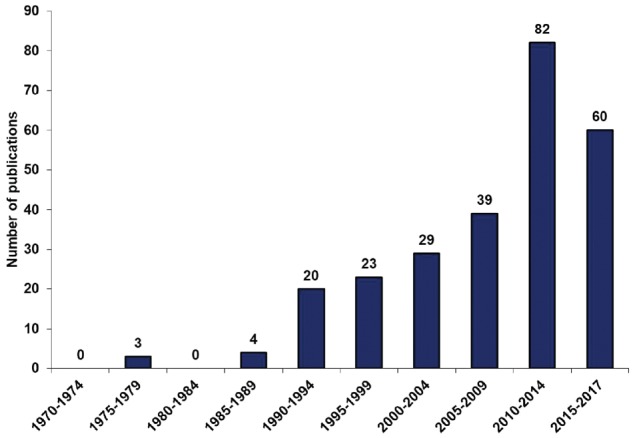
The number of included studies on the birth prevalence of CHD in five-year bands from 1970 to June 2017. Year-bands are defined according to the publication time of included studies.

A total of 260 studies were included in the meta-analysis according to the outlined review process in Figure 1. These studies were of satisfactory quality with a mean NOS score of 7.29 (range 4–9). Among studies reporting the method of diagnosis (54.6%), 95.8% utilized echocardiography. Of the studies included, 142 (55%) had been published since 2010 (Figure 2); among the post-2010 studies, 32 had a sample size greater than 1 million births, whereas this had been the case for only five studies published prior to 2009. Of these post-2010 studies, 99 were reported from Asia (49) and Europe (50) and 36 were from North America. In sum, 103 732 049 live births were included from the post-2010 studies, whereas 27 026 802 births were collected from studies published in 1970–2009.

**Figure 3.**
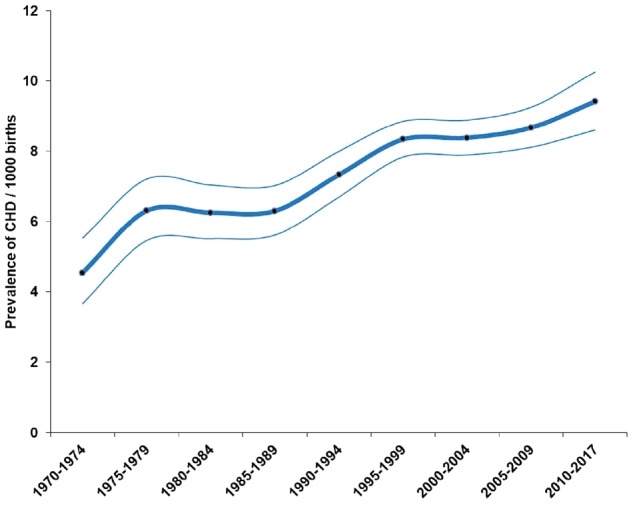
Changes in the birth prevalence of CHD 1970–2017. The thick line is the estimated overall prevalence of CHD, thin lines represent the 95% CI.

A total of 130 758 851 live births (1 161 030 CHD cases) were included in the analyses. The mean prevalence of CHD 1970–2017 globally was 8.224 (7.817, 8.641) per thousand. There was no significant difference between the prevalences estimated from hospital-based studies and from national registry studies (*P* = 0.118). Time trend analyses indicated a rise from 4.547 (3.664, 5.526) per thousand in 1970–74 to 9.410 (8.602, 10.253) per thousand in 2010–17 (*P* < 0.001). Previous analyses had indicated an apparent plateau in global CHD prevalence between 1995 and 2009; however, by comparison with these years there was a ∼10% increase in CHD prevalence from 2010–17 (*P* = 0.031) (Figure 3).

**Figure 4.**
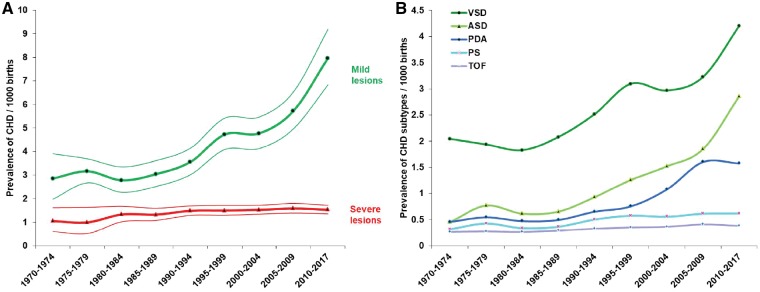
The birth prevalence of CHD subtypes and their changes over time. (A) The prevalence of mild and severe CHD lesions during 1970–2017. Thick lines are the estimated prevalence of CHD lesions, thin lines represent the 95% CI. (B) The birth prevalence of the five most frequent CHD subtypes during 1970–2017. PS, pulmonary stenosis; TOF, tetralogy of fallot.

VSD, ASD and PDA (the ‘mild’ lesions) were, as anticipated, the three most frequent types of CHD (Table 1). In total, these mild lesions contributed 57.9% (54.9, 61.0) to the total burden of CHD, with an increasing trend from 49.2% (43.4, 54.9) in 1970–74 to 65.3% (58.7, 71.7) in 2010–17 (*P* < 0.001). During the review period, the reported prevalence of these lesions had increased almost 3-fold, in contrast with a much less marked increase among severe lesions (Figure 4A). The most marked estimated increase in prevalence among the mild lesions was for ASD (Figure 4B), which increased 6-fold from 0.452 (0.339, 0.582) per thousand in 1970–74 to 2.858 (2.076, 3.764) per thousand in 2010–17 (*P* < 0.001). Changes in prevalence of VSD, ASD and PDA together explained 93.4% of the increase in prevalence of total CHD.

**Table 1.** The prevalence and percentages of 27 CHD subtypes

CHD subtype	Prevalence of CHD subtype per thousand (95% confidence interval)	Percentage of CHD subtype, % (95% confidence interval)
Ventricular septal defect	3.071 (2.845–3.305)	35.568 (33.876–37.278)
Atrial septal defect	1.441 (1.215–1.687)	15.378 (13.492–17.363)
Patent ductus arteriosus	1.004 (0.803–1.228)	10.172 (8.519–11.954)
Pulmonary stenosis	0.546 (0.485–0.611)	6.233 (5.703–6.784)
Tetralogy of Fallot	0.356 (0.326–0.387)	4.422 (4.064–4.794)
Transposition of the great arteries	0.295 (0.269–0.322)	3.819 (3.446–4.210)
Atrioventricular septal defect	0.290 (0.265–0.316)	3.595 (3.302–3.900)
Coarctation of the aorta	0.287 (0.261–0.314)	3.570 (3.273–3.879)
Pulmonary arteriovenous aneurysm	0.272 (0.153–0.425)	2.975 (1.858–4.343)
Congenital heart block	0.268 (0.028–0.752)	3.223 (0.268–9.263)
Aortic valve insufficiency	0.251 (0.137–0.400)	2.318 (1.271–3.667)
Aortic stenosis	0.186 (0.161–0.214)	2.334 (2.016–2.674)
Hypoplastic left heart syndrome	0.178 (0.162–0.195)	2.564 (2.251–2.897)
Mitral insufficiency	0.152 (0.097–0.220)	1.348 (0.899–1.886)
Tricuspid atresia or stenosis	0.117 (0.091–0.146)	1.071 (0.905–1.250)
Double outlet right ventricle	0.106 (0.090–0.124)	1.303 (1.127–1.491)
Pulmonary atresia	0.098 (0.085–0.112)	1.308 (1.113–1.518)
Single ventricle	0.093 (0.080–0.108)	1.145 (0.975–1.330)
Dextrocardia	0.089 (0.073–0.106)	1.027 (0.825–1.250)
Total anomalous pulmonary venous return	0.083 (0.071–0.095)	1.501 (1.163–1.882)
Mitral stenosis	0.083 (0.047–0.130)	0.955 (0.564–1.446)
Truncus arteriosus	0.078 (0.067–0.089)	0.982 (0.849–1.124)
Ebstein anomaly	0.044 (0.040–0.049)	0.534 (0.467–0.606)
Coronary artery aneurysm	0.044 (0.025–0.068)	0.417 (0.287–0.571)
Interrupted aortic arch	0.041 (0.032–0.051)	0.609 (0.412–0.844)
Partial anomalous pulmonary venous return	0.039 (0.027–0.053)	0.314 (0.238–0.400)
Cor triatriatum	0.022 (0.014–0.031)	0.245 (0.125–0.405)

**Figure 5.**
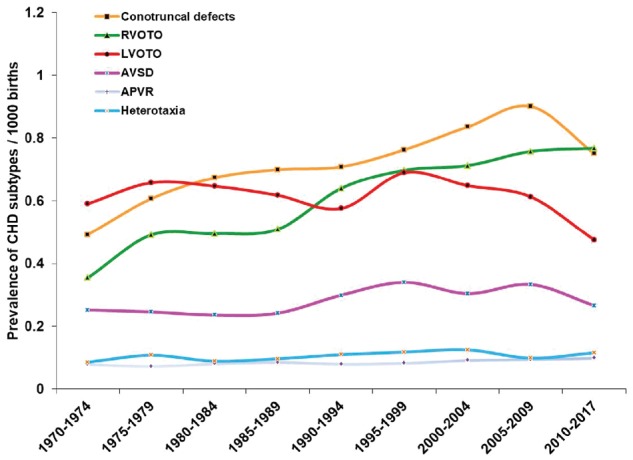
The changes in birth prevalence of CHD categories (except septal defects) from 1970 to 2017. RVOTO, right ventricular outflow tract obstruction; LVOTO, left ventricular outflow tract obstruction; AVSD, atrioventricular septal defect; APVR, anomalous pulmonary venous return.

Trends in birth prevalence of CHD lesions other than septal defects, classified according to Botto’s approach, are shown in Figure 5. Two trends apparent from these data are: (1) a progressive increase in the estimated prevalence of right ventricular outflow tract obstruction (including infundibular, pulmonary valvar, supravalvular and pulmonary arterial defects), which approximately doubled from 0.355 (0.189, 0.572) per thousand to 0.767 (0.615, 0.935) per thousand during the study period (*P* = 0.001); and (2) a decrease by approximately one-third in the estimated prevalence of left ventricular outflow tract obstruction (LVOTO, which includes hypoplastic left heart syndrome) from the mid-1990s onwards – from 0.689 (0.607, 0.776) per thousand in 1995–99, to 0.475 (0.392, 0.565) per thousand in 2010–17 (*P* = 0.004 for two-group comparison; *P* = 0.023 for decreasing trend 1995–2017).

**Figure 6.**
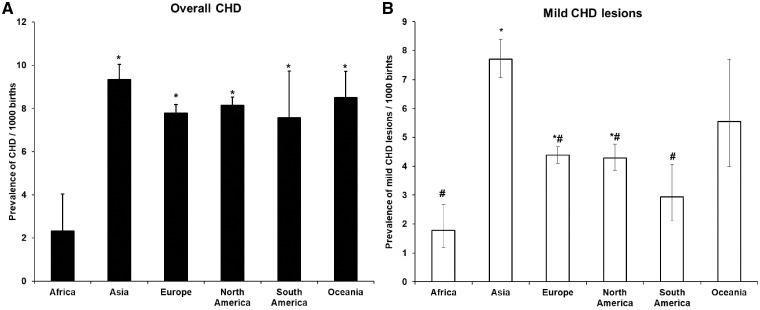
CHD prevalence in different geographic regions 1970–2017. (A) The prevalence of overall CHD in six geographic regions. (B) The prevalence of mild lesions in six geographic regions. The number of studies for each region was: Africa 4 (69 304 births), Asia 74 (12 975 858 births), Europe 110 (56 272 142 births), North America 58 (59 498 436 births), South America 9 (667 353 births) and Oceania 5 (1 275 758 births). Data for (A) and (B) are presented as Mean ± SE. *, *P* < 0.05, compared with Africa, #, *P* < 0.05, compared with Asia.

Africa was the region with the lowest CHD prevalence reported for 1970–2017 among the six global regions analysed (*P* < 0.001); CHD prevalence in Africa was estimated as around a quarter of that in other parts of the world (Figure 6A). Although additional data from Africa were available since 2010 (three post-2010 studies) it remained very sparse. There were no differences between prevalences among the other five regions (Figure 6A). When analysis was restricted to mild lesions only, the prevalence in Asia was significantly higher than elsewhere in the world (*P* < 0.001) (Figure 6B). In addition, the rate of increase of CHD prevalence (ASD in particular) in Asia (33.0% every 5 years) was greater than in other regions (*P* = 0.003; Supplementary Figure 1, available as Supplementary data at *IJE *online).

**Figure 7.**
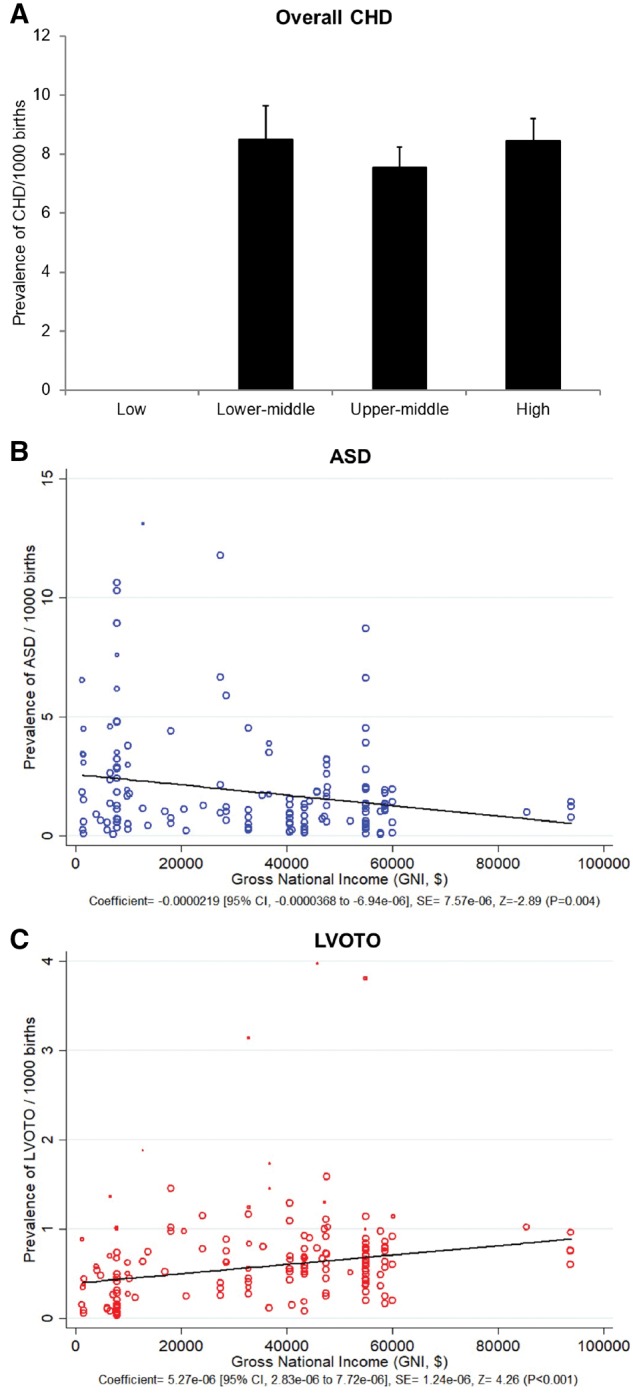
CHD prevalence and income levels. (A) The prevalence of CHD in countries of different income levels. (B) The linear correlation between the prevalence of ASD and GNI (per capita). (C) The linear correlation between the prevalence of LVOTO and GNI (per capita).

Insufficient data were available to conduct analyses relating to national income in countries of ‘Low’ level. There was no significant difference in CHD prevalence between countries of ‘High’, ‘Higher-middle’ and ‘Lower-middle’ income levels (Figure 7A). Intriguingly, the prevalence of ASD showed reverse correlation with gross national income (GNI) (*P* = 0.004) (Figure 7B), such that ASD appeared more prevalent in countries with lower national income, whereas the prevalence of LVOTO was higher in countries with higher national income (*P* < 0.001) (Figure 7C). 

## Discussion

Compared with the comprehensive meta-analysis to 2010 conducted by van der Linde *et al.*,^1^ the present study of the literature to 2017 involves more than twice the number of studies and over five times the number of births. It incorporates a number of reports from surveillance registries of greater than 1 million people since 2010. This enabled us to more precisely estimate prevalence trends across the world and to carry out more in-depth analyses of CHD phenotypic subgroups.

Overall between 1970 and 2017, the prevalence of CHD globally increased by 10% every 5 years, with over 90% of this increase probably due to increased detection of milder lesions (VSD, ASD and PDA).^12^ The single lesion with the greatest increase in reported prevalence was ASD, which showed a 6-fold increase when the period 2010–17 is compared with 1970–75. In agreement with the previous meta-analysis of van der Linde *et al.*,^1^ we found a relative stabilization of the overall prevalence of CHD in 1995–2009. However, when data published up to 2017 is incorporated, a further upward trend in prevalence is evident since 2009. Moreover, the stability of CHD prevalence in the years 1995–2009 masked some opposing trends for individual lesions, which with our large dataset we were able to distinguish; during this period the prevalence of ASD and PDA increased substantially whereas the prevalence of LVOTO decreased significantly. The fall in prevalence of LVOTO – which includes severe lesions such as hyoplastic left heart syndrome and aortic atresia – would be consistent with higher availability of prenatal echocardiography and the choice of termination of pregnancy in the presence of such severe lesions worldwide, as previously documented in single countries, for example, by Hunter *et al.*^13^

Greater use of echocardiography globally, and improved echocardiographic techniques, are likely to account substantially for the increased prevalence of mild lesions. There was heterogeneity in the CHD prevalence changes in different regions of the world, with Asia showing the most marked rise in prevalence of mild lesions. This would be in keeping with rapid improvements in socioeceonomic status and healthcare availability in these countries.^14^^,^^15^ However, our data suggest this may not entirely account for the trends observed. In the most recent time period, the prevalence of ASD in Asia was higher than in European countries and the United States, which are high-income countries where the diagnostic rate of ASD would be anticipated to be at least equal to that in the largely middle-income countries of Asia. Moreover, globally the prevalence of ASD was significantly negatively correlated with GNI. The data therefore indicate a potentially higher exposure to genetic or environmental factors predisposing to ASD in lower-income countries, particularly in Asia, than in high-income Western countries. One possible candidate is air particulate matter (PM) pollution. PM is already known to have adverse cardiovascular consequences in adults,^16^ and recent studies indicate that maternal exposure to PM may be a risk factor for septal defects in particular.^17–19^

The reported prevalence of CHD in Africa continues to be significantly lower than in other regions of the world. However, data from Africa were sparse – only four studies were eligible for inclusion in this meta-analysis. It seems very likely that inadequate access to healthcare resources, leading to a low detection rate, accounts for the discrepancy in prevalence between Africa and the rest of the world, although true differences in prevalence due either to different incidence of CHD *in utero*, increased rate of miscarriage for affected pregnancies or poorer survival in the immediate neonatal period are not ruled out. Our data confirm previous findings that resources devoted to the care of patients with CHD in Africa are inadequate, and that both further research and improved access to care are required on the African continent.

This study has limitations. Grouping of lesions may not have always reflected their severity – for example, large VSDs are severe lesions which left untreated can lead to Eisenmenger’s syndrome.^20^ We were unable to distinguish sub-phenotypes of VSD or other lesions, which could have led to an underestimate of severe lesions. The application of different versions of International Classification of Diseases (ICD) codes in included studies may have a weak influence on the recording of CHD subtypes. The codes for CHD subtypes in this analysis are typically a one-to-one correspondence between ICD-9 and ICD-10 (version presently applied), with two exceptions. Eisenmenger’s syndrome is falling in the group of VSD in ICD-9 but not in ICD-10. This situation would be highly unlikely to influence the records of VSD for births, since Eisenmenger’s syndrome presents later in life. The code ‘Malposition of heart and cardiac apex’ in ICD-9 refers to dextrocardia and laevocardia, which are coded separately in ICD-10. The studies with ICD-9 codes could therefore possibly result in an overestimation of the prevalence of dextrocardia. The studies included in the meta-analysis showed high heterogeneity, as expected, given the unknown aetiological factors for around 80% of CHDs.^2^ Potentially, hospital-based studies could have provided upwardly biased estimates of prevalence compared to national registries, both of which were included in the meta-analysis; however, we found no evidence of heterogeneity in the overall prevalence reported between these two subgroups of studies. Moreover, hospital-based studies are particularly valuable in countries where national registry data are not available. It is known that genetic copy number variants and *de novo* single-nucleotide variants may account for up to 20% of CHD accompanied by extracardiac malformations and/or intellectual impairment.^21^ We were unable to stratify participants in the individual studies based on these additional diagnoses. Finally, studies generally had very little data available about potential environmental influences on CHD, limiting our capacity to explore the dataset for environmental associations.

In conclusion, we have shown a continued increase in the reported birth prevalence of CHD since 2009, which has important implications for healthcare resource allocation, particularly in developing countries. The rate of ASD now appears higher in Asia than in Western countries. Prenatal diagnosis and consequent termination of pregnancy may have accounted for a reduction in the rate of LVOTO worldwide, particularly in richer countries. Prevalence rates in Africa remain significantly lower than elsewhere in the world, highlighting the need for additional healthcare resources and research studies in African patients with CHD.

## Funding

The British Heart Foundation (Personal Chair award to BK), The University of Manchester-Peking University Health Science Centre Alliance, China Scholarships Council, UK Medical Research Council, and South African Medical Research Council.

**Conflict of interest**: None declared.

## Supplementary Material

Supplementary Data
