# Supplementary material for: Global birth prevalence of congenital heart defects 1970–2017: updated systematic review and meta-analysis of 260 studies
Source: Int J Epidemiol. 2019 Feb 19;48(2):455–63. doi: 10.1093/ije/dyz009 (PMC6469300; doi:10.1093/ije/dyz009)
Supplement: Supplementary Data [file dyz009_supp.zip › dyz009-Suppl_data/dyz009_Suppl_1.docx]

Supplementary figures and tables


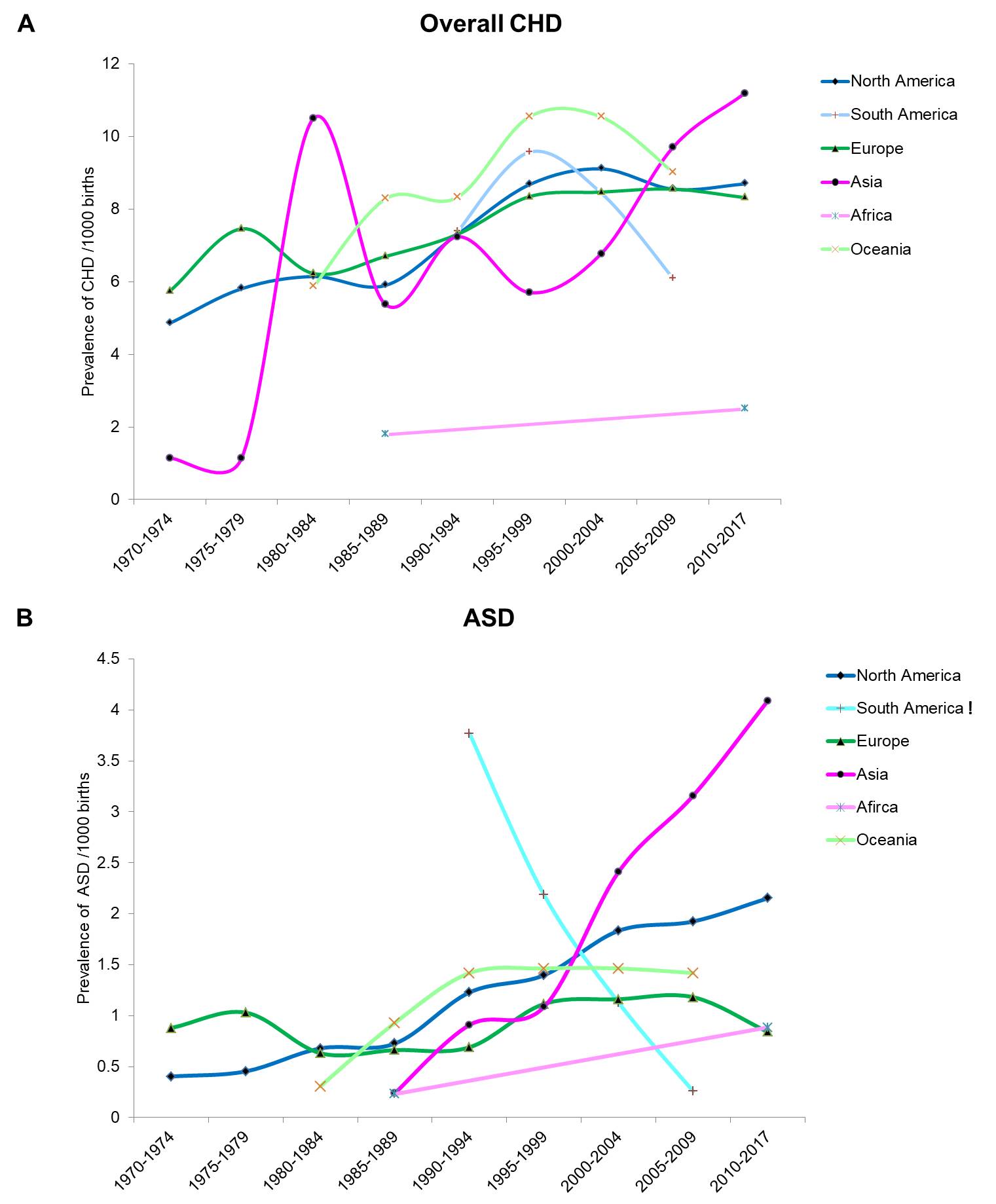


Figure S 1 The changes of CHD prevalence and ASD prevalence in different geographic regions. The increasing rates of prevalences of overall CHD (A) and ASD (B) in Asia were faster than other regions, p<0.05. !, the result was not reported in the main text; data for ASD in South America was only reported in 6 studies from Brazil, Colombia and Educator, which was insufficient to interpret the trend in South America.

Table S 1 The including and excluding criteria for paper review

| **Including criteria** | **Excluding criteria** |
| --- | --- |
| - The subjects were human beings. | - Studies on animals. |
| - Studies were conducted with a focus on the prevalence of CHD. | - Studies did not report on overall CHD prevalence or focus on severe or other CHD subgroups only. |
| - Papers should report the total number of CHD cases and sample sizes, or these numbers could be calculated from the reported data. |  |
| - Studies were original papers and published in peer-reviewed journals with English abstracts. | - Papers of reviews, comments, books or editorial. |
|  | - Results were non-original but estimation from other data sources. |
|  | - No English abstracts. |
|  | - Conference abstracts. |
| - Sampling from natural or unselected populations. | - Studies about the prevalence of CHD in special populations, like Down syndrome population, offspring from mothers with Diabetes, Children with other defects, twins, or other situations with known factors which can change the risk of CHD. |
| - The investigation time of studies was not earlier than 1970. | - Studies conducted before 1970. |
| - The population was aging of 0-6, which is adapted from the age criteria of many birth defects surveillance registries. | - Studies on adults or children with the age > 6 years old |

Table S 2 Classification of CHD subtypes based on Botto’s method (2007)

| Classification | Subtypes | ICD10 Code |
| --- | --- | --- |
| Heterotaxia | Dextrocardia | Q24.0 |
| Conotruncal defects | Truncus arteriosus, Interrupted aortic arch, D-transposition of great arteries, Tetralogy of Fallot, Double outlet right ventricle | Q20.0, Q25.1A, Q25.2, Q25.3, Q25.4, Q20.3, Q21.3, Q20.1 |
| AVSD | Atrioventricular septal defect | Q21.2 |
| APVR | Total and Partial anomalous pulmonary venous return | Q26, Q26.2, Q26.4, Q26.8, Q26.9 |
| LVOTO | Hypoplastic left heart syndrome, Coarctation of aorta, Aortic stenosis | Q23.4, Q25.1, Q23.0 |
| RVOTO | Pulmonary valve stenosis only, Tricuspid atresia, Ebstein anomaly, Pulmonary artery atresia | Q22.1, Q22.4, Q22.5, |
| Septal defects | Ventricular septal defect only, Atrial septal defect only, Ventricular septal defect and Atrial septal defect only | Q21.0, Q21.1, (Q21.0 and Q21.1) |

Table S 3 Classification of severe CHDs based on Hoffman's method (2002) and modified according to methods from Alexander (2014) and Raluca (2009).

| Severe CHD | Subtypes |  | ICD 10 Code |
| --- | --- | --- | --- |
| Cyanotic lesions | 1. D-transposition of the great arteries | | Q20.3 |
|  | 2. Tetralogy of Fallot | | Q21.3 |
|  | 3. Right heart lesions  a. Tricuspid atresia  b. Pulmonary atresia  c. Ebstein anomaly | | Q22.4  Q22.0  Q22.5 |
|  | 4. Hypoplastic left heart syndrome  5. Interrupted aortic arch | | Q23.4  Q25.2 |
|  | 6. Single ventricle | | Q20.4 |
|  | 7. Double outlet right ventricle | | Q20.1 |
|  | 8. Truncus arteriosus | | Q20.0 |
|  | 9. Total anomalous pulmonary venous connection | | Q26.2 |
| Acyanotic lesions | Atrioventricular septal defect | | Q21.2 |
